# Some Observations on the Pathology and Prevailing Diseases of Warm Climates

**Published:** 1804-03-01

**Authors:** A. Pearson

**Affiliations:** Member of the London College of Surgeons, and Surgeon in the Service of the East India Company


### *Some* Observations *on the* Pathology *and* prevailing Diseases *of* Warm Climates; *by* A. Pearson, *Member of the London College of Surgeons, and Surgeon in the Service of the East India Company.*

( Continued from our last pp. 158—168. )

THE ventilation, which hot climates permit and demand, is a powerful cause why people in them are less liable to the contagious forms of febrile disease; I place the typhus icteroides out of the question, having had no opportunity of seeing that disease. In the early part of a passage from this country to India, and in crowded ships, a fever arises from the accumulation and effect of human miasmata, which is to be regarded as the typhus navium, and it in general corresponds to the nosological definition of that disease.

disease. In some cases, there is much more heat and vascular action than the definition supposes, and in others we find cough, and some degree of pneumonic affection. Writers on this subject, mention a peculiar burning sensation (calor mordicans) excited on the fingers by feeling the pulse as a diagnostic; where I have had opportunities of observing the disease, I have not been sensible of this symptom. The treatment best adapted, seemed to be sponging the body, and especially the lower extremities, with cold or tepid vinegar and water; an emetic at the commencement; laxatives pretty frequently, but not so as to induce full purging; spt. Mindereri with spt. ætheris, vitriolici seu nitrosi, decoction of bark with confectio cardiaca & vitriolic acid; 5 drops of tincture of opium, given every second hour alternately with a glass of port wine; anodyne draughts; blisters to the breasts and thighs; decoction of bark and serpentaria; a cordial diet as soon as the powers of digestion are recovered. No preventive means ought to be omitted; these consist of frequent and effectual ventilation by windsails, and opening the ports of the ship; by getting up the hammocks daily; by cleanness and dryness of the decks; by personal cleanliness; by not allowing the people to remain below when the ships are crowded with soldiers, not even at meals; by fumigation with nitrous fumes, as pointed out by Dr. Smyth, by white washing, and sprinkling with vinegar.

In the warm climates, the attacks of febrile disease are generally accompanied with symptoms of bilious absorption, and torpor of the intestinal canal, and with a greater or less tendency to remission.

The treatment recommended by authors is very contradictory; some advising a continued and severe evacuant plan, while others administer bark on every appearance of remission, and even without waiting for any. If purging with calomel and neutral salts is assiduously practised in the first days, giving intermediately mild diaphoretic and antimonial medicines, the use of bark will be found unnecessary, or may be entered on with much greater safety and advantage. I am doubtful if the genuine remittent fever appears without a previous exposure to the exhalation of marshes, or that from rank vegetation; and the distinct remissions and exacerbations described in books, are not frequently to be met with. In remarking upon this, I will consider it as I have seen it at Bencoolen, which is an unhealthy situation; and the disease as it appears there is, I believe, the endemic of Batavia in general, answering to the the nosological definition of the tritæophya paludosa, frequently however with a shorter remission, the apyrexia being in different cases more or less complete. It is frequently some time after the application of the remote causes before the disease comes on, as is proved by its not appearing in a ship which has received it there, frequently a fortnight after leaving the port. The peculiarity of its attack is in the symptoms of determination to, and congestion in the head, the throbbing of the temples, flushing of the face, hurry and incoherence of thought. It is in this state that the rapid dissolution which sometimes follows the attack takes place, otherwise the disease will run out to a considerable length, and even for years, with intervals of imperfect convalescence, the complaint then carrying off the patient during a cold fit, or by an attack of dysentery, or rather of colliquative diarrhœa. The other symptoms are nearly as detailed in books. The debilitating effect of the marsh miasmata is generally recognized, (the sedative effects of hydro-carbonate gas, proved to form a considerable part of them, is well known) and it is probable that the nervous energy and muscular irritability, are much and suddenly impaired by their impression upon the sensorium; the powers of circulating the mass of blood are for a time diminished; from that, and irregular actions of the vessels of different viscera, a relative degree of plethora and inflammation takes place, while, from the excretories being similarly affected, the power which the economy possesses to rid itself of an excess of heat is abated. In such a state, it is not surprising that congestions should take place in the brain and glandular viscera, and such symptoms of nervous and vascular excitement, as above related, be the consequence.

This attempt to state the operation of remote and proximate causes is made principally with a view to the treatment, and to inculcate caution as to the means generally resorted to; I mean, in the use of general blood-letting, large and frequently repeated doses of stimulant purgatives, and affusion of cold water. It is probable that less hazard will be incurred, and greater benefit derived, from an immediate restriction to the antiphlogistic regimen in all its branches, especially in abstraction of the stimuli of light, heat, and exercise, in a free but guarded use of calomel and saline purgatives, in sponging the body with cold or tepid vinegar and water. The disease however, in this violence of symptoms, is not very frequent, and it is most commonly seen as described in in books. At the commencement to avoid emetics, to procure free evacuations of the bowels by calomel, neutral salts, inf. sennæ, c. tart. solub. and when the calomel affects the mouth, the disease frequently disappears. During the first days, and at all times during the hot stage, nitrous, camphorated, or the common saline medicines with antimonial wine. An early recourse ought be had to the use of the bark during the remissions, but it will not be found to be so generally admissible or efficacious as some publications would lead us to expect. Where there is increased biliary secretion, irritability of the stomach, or an obstructed state of any of the viscera, it will be rejected, and the beneficial effects from it precarious. It is proper, however, to give it a trial under different forms when the substance disagrees, and the infusion or decoction may be made trial of along with the tincture, extract snake-root, angustura, vitriolic acid, or other combinations. In whatever form, the doses ought to be as large and frequent as the stomach will bear, during the remission only, and joining it with such a portion of p. rhæi, magnesia vitriolata, or other laxative, as may produce two stools in the 24 hours. More effectual doses of calomel and other laxatives may be given from time to time. In case of the disease resisting these means, small doses of calomel, with or without opium, and continued for some length of time, may be had recourse to. Where there was much retching, fomentations, blisters, and cataplasms, applied to the pit of the stomach, and the saline effervescing draughts, are of use; and where the debility is so great, that danger is apprehended from each returning paroxysm, an opiate given in warm wine will sometimes prevent the accession, and interrupt the morbid habit.

The use of opiates however in these affections, or indeed in any of the diseases occurring in European constitutions in hot climates, is not generally beneficial, nor to be adopted but with discrimination. As there are very different states of the constitution at different periods of the progress of the disease, the regimen is to be adapted to these as they occur; the antiphlogistic, nutritious, and cordial plan of diet must be had recourse to in their turns. The intermittent fevers are occasionally met with in warm climates, and are more prevalent in China than in any other part of the East; there the summer and autumnal heats are succeeded by cool and wet weather; the face of the country is flat, marshy, and subject to be overflowed. The customary practice is followed in the treatment of them. An

 emetic emetic given at the commencement, and repeated from time to time in the course of the disease, laxatives, diminishing the dose and frequency of their administration as it advanced. The bark in different forms and combinations, given as early and as copiously as possible during the apyrexia; and as in such situations, torpor of the intestinal canal and bilious secretions are not unusual, from one to four grains of p. rhæi, or ℨj. of tinctura aloës added to each dose will be proper. Alterative doses of calomel, flores zinci, chamomile flowers with ferrum ppt. in the form of an electuary, were tried in their turns, and found to be useful. Of the effects of arsenic I have no experience. With regard to the compression of arteries with tourniquets, it has appeared to me that where it was early had recourse to, and the paroxysms were stopped by it, a dysenteric action of the bowels was apt to supervene; where the disease was of longer standing, it was extremely useful by interrupting the habit of morbid action, without any such unfavourable consequences.

It may be proper to advert here, to what is called the lunar influence; and however unaccountable the connection between that and the human frame may be, and however sceptical the mind regarding it, a careful observation of diseases in these climates will corroborate the inferences of Dr. Balfour, that the attacks and fatal terminations of febrile disease and of dysentery, a disposition to contraction and retention in the intestinal canal, aggravations of spasmodic and nervous affections take place most frequently during the lunar periods, *i. e.* in 50 hours before and after each new and full moon; he points out diurnal and quarterly effects of the same kind not so observable, as well as equinoctial periods.

What are called bilious attacks, are frequently to be met with in warm climates, and are often perplexing to the young practitioner, to whose mind (if he does not attend to the state of the pulse, which is but little altered) a loose analogy will present the idea of a violent fever from the retching, yellow suffusion of the face and eyes, whiteness of the tongue, general perturbation and irritability. It is to be treated at first by laxatives and gentle diaphoretic medicines, and a liquid abstemious regimen; after the first two or three days, calomel, and the more powerful cathartics may be given.

The purely inflammatory diseases of warm climates are few in number; while in the European constitution, those of a mixed inflammatory description are not unfrequent; this this is proved by dissections in different epidemics, where an inflamed state of the stomach, of the colon, membranes of the liver, &c. have been detected.

The hepatitis is the inflammatory disease most frequently met with; but a considerable variety of modifications, and very different states of hepatic affection existing under that name, it will be useful to attempt at least to discriminate them. When a constitution such as has been before described, of plethora and accumulated excitability, is first subjected to the stimulus of heat, frequently at the same time the organ itself is locally excited by the application of animal food, spirituous liquors, and other acrid ingesta, with only the intervention of the coats of the stomach, it is not surprising that active inflammation should take place. This is denoted by severe pain in the right hypochondrium, febrile affection, cough, pain at the top of the shoulder, retching, &c. This once induced will be apt to recur as inflammations in other parts, and may terminate in suppuration; but the very acute form of the disease is not that which is generally met with. A morbid sensibility, and readiness of the organ to take on inflammatory action; a want of activity and muscular power in the branches of the vena portarum, giving rise to congestion, and an irregular and vitiated secretion and discharge of the bile; induration of the glands, and obstruction of the vessels, approaching to scirrhus, or actually in that state; and these combined with symptoms of impaired digestion, loss of tone, and increased incitability of the system, are the most frequent varieties of hepatic affection.

We have no accurate diagnosis, by which to distinguish between affection of the liver arising from the above causes, and that which is connected with scrophulous tubercles; nor yet from the mere enlargement of the organ without morbid alteration of its substance or structure, which frequently I believe takes place, when the power of the absorbents in renewing solid parts is diminished; or even from the symptoms produced by a temporary congestion, either in the organ itself or the adjacent viscera, as the stomach, or ascending colon. We are in a similar difficulty to ascertain whether suppuration has taken place or not; it has frequently been found to exist where it had not been suspected; and, on the other hand, where there were several symptoms to denote it, it has not been found. From the matter being shut up, the more decided symptoms of hectic are wanting, and quickness of pulse, irregular febrile accessions, and flushing, are so often attendants of the irritable ritable state brought on by residence in hot climates, that they afford no certain criterion. An obtuse pain in the right hypochondrium is mentioned as being a symptom; but this occurring in all the different states of the complaint, often where there is no hepatic disease, must be a fallacious one. Its being so generally stated by authors to be a diagnostic, has the unfavourable effect of alarming the apprehensions of those who have morbid sensations in that part, and who are in general sufficiently prone to indulge exaggerated feelings or fears on the subject of their health or sensations.

It is obvious, that in all these varieties of disease the same mode of treatment cannot be adviseable; and with a view to practice, it must be ascertained under which of the states the symptoms appearing are to be classed. If under the circumstances, and with the symptoms of active inflammation, general blood-letting is adviseable, and safe to a greater extent than is commonly practised, locally also by cupping or leeches; fomentations, blistering over the seat of the pain, and dressing the blister with mercurial ointment; above all, by mercurial purges given in repeated doses, as calomel from two to four grains every two hours till purging is produced; and when the state of the stomach permits, neutral salts, otherwise the calomel continued; a very dilute solution of tartarized antimony, but never so as to excite vomiting; a total abstraction of the stimuli of light and muscular action, and of heat as far as that can be affected; a diet diluent and vegetable; and these means repeated or continued till the severity of the inflammation is abated. Some torpor or obstruction of the circulating vessels, and pori or ductus biliarii, have, it is probable, preceded this attack of inflammation; at any rate, after the excessive action, they are left in such a state, or in one very ready to assume it. It is then that we may expect the greatest benefit from a mercurial course, conducted by friction of the ung. hydrargyri upon the right side, the blue pill taken by itself, or united with calomel; calomel occasionally in purgative doses; these are to be employed so as to produce some spitting, and the mercurial action kept up by the same means, for weeks or months afterwards; at first, rather a spare diet, and afterwards a more nutritious one; to abstain from stimulating drinks, to use horse exercise, and latterly, but with much caution, tonics or chalybeates, accompanying them with the use of laxatives.

These comprehend the greatest part of the resources in the treatment of chronic hepatitis, or schirrus, and the state state of the circulating and secretory vessels, which leads to these complaints. When these fail, a removal to the native or a more northern climate becomes necessary; and from this state there are frequent recoveries effected by such a change; but a residence of a complete year is in general requisite before the feelings are pleasurable, or the actions of the economy are restored to their healthful state, and then it is probable that the benefit accrues, when the obstruction is of that kind to admit of being removed by the efforts of the system in the gradual absorption and renewal of solids.

As before mentioned, the diagnostics of the different species of hepatic affection are not well marked. The existence of scrophulous tubercle giving rise to the symptoms of it must be inferred from observations on the habit, and in it, as well as confirmed scirrhus and hepatalgia, or pain seated in the organ, or right hypochondrium, which have resisted repeated mercurial courses, it is best not to attempt by a long protracted use of this medicine to remove it. The irritation and debility induced by it, do more than counterbalance the deobstruent effects of its stimulant operation. A regimen, such as has been before recommended, nutritious, but not stimulant or viscid, taking care not to overload the organs, a total abstinence from vinous, spirituous, or fermented liquors, horse exercise, and even cold bathing. Bitters, tonic and chalybeate medicines, given so combined with laxatives, as p. rhæi or aloes, and perhaps natron, as to be to the intestinal canal a stimulus, such as that which the bile affords, and which in such a state is sparingly and imperfectly secreted; blisters applied to the side; a discharge kept up by issues, or setons placed near to the situation of the pain, will likewise be found useful. In this state also the patient cannot expect a cure, or even effectual relief, but from returning to a colder climate; nor then, but from a long continued and careful observance of the plan of regimen and medicine pointed out.

